# Mitochondrial Transplantation Rejuvenates Aging Heart by Restoring Mitophagy Flux via the HIF‐3α‐BNIP3 Axis

**DOI:** 10.1111/acel.70720

**Published:** 2026-09-23

**Authors:** Ning Jin, Li Zhou, Haixia Gui, Jingjing Tang, Xiaozhen Huang, Wenting Wang, Guorong Jin, Haiqin Cheng, Yuxiang Liang, Xinyi Geng, Zhiwei Peng, Hong Zhao, Zhizhen Liu, Jun Xie

**Affiliations:** ^1^ Department of Biochemistry and Molecular Biology, Shanxi Key Laboratory of Birth Defect and Cell Regeneration Shanxi Medical University Taiyuan China; ^2^ Key Laboratory of Coal Environmental Pathogenicity and Prevention (Shanxi Medical University) Ministry of Education Taiyuan China; ^3^ Department of Cardiology The Second Affiliated Hospital of Shanxi Medical University Taiyuan Shanxi China

**Keywords:** aging heart, HIF‐3α‐BNIP3 axis, mitochondrial transplantation, mitophagy flux

## Abstract

Mitochondrial quality control is severely impaired in the aging heart, largely attributed to disrupted mitophagy homeostasis. However, the key molecular drivers remain poorly defined, and the translational value of mitochondria‐targeted therapy for cardiac aging is still underexplored. Here, we report prominent mitophagy flux congestion in aged cardiac tissue and confirm that mitochondrial transplantation efficiently rescues impaired mitophagy, ultimately rejuvenating the aging heart. Mechanistically, we identify a novel HIF‐3α‐BNIP3 signaling axis in the aging heart: HIF‐3α, conventionally recognized as a transcriptional repressor, is aberrantly upregulated in senescent cardiomyocytes and directly regulates excessive BNIP3 expression to trigger mitophagy congestion. Notably, we establish an innovative translational strategy that mitochondrial transplantation restrains pathological overactivation of the HIF‐3α‐BNIP3 axis via improving intracellular ATP homeostasis, thereby reconstructing normal mitophagy flux and reversing cardiac aging. Our findings uncover an unrecognized upstream regulator of age‐related mitophagy defects and provide a mitochondrial‐based intervention approach for the treatment of aging‐associated cardiac dysfunction.

## Graphical abstract

Aging heart shows mitophagy flux congestion and mitochondrial dysfunction. Aberrant activation of the HIF‐3α‐BNIP3 axis induces mitophagy flux congestion in the aging heart. Mitochondrial transplantation rectifies the HIF‐3α‐BNIP3 axis and restores mitophagy flux by elevating ATP production in the aging heart.

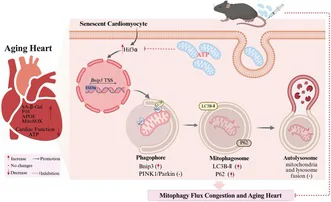

**Abbreviations**
AHA: American Heart Association
APOE: apolipoprotein E
BMSCs: bone mesenchymal stem cells
BNIP3: BCL2‐interacting protein 3
DEGs: differentially expressed genes
EF: Ejection Fractional
FBS: fetal bovine serum
FS: fractional shortening
GSEA: Gene Set Enrichment Analysis
HIF‐1α: hypoxia‐inducible factor 1 alpha
HIF‐3α: hypoxia inducible factor 3α
HPA: Human Protein Atlas
KEGG: Kyoto Encyclopedia of Genes and Genomes
LIR: LC3‐interacting region
LVEDV: left ventricular end‐diastolic volume
LVESV: left ventricular end‐systolic volume
MitoSOX: mitochondrial superoxide
OE: overexpression
PMSF: phenyl methane sulfonyl fluoride
RNA‐Seq: RNA sequencing
ROS: reactive oxygen species
TEM: transmission electron microscopy
TFs: transcription factors
WB: western blot
WGA: Wheat Germ Agglutinin

## 1 Introduction

As population aging accelerates worldwide, the burden of cardiac aging related diseases is increasing, and has become one of the main causes of death and disability among the elderly. According to the American Heart Association (AHA) 2025 report, both the prevalence and mortality of cardiovascular diseases continue to rise, and the prevalence of cardiovascular related diseases is significantly positively correlated with age growth (Martin et al. 2025; Ruiz‐Garcia et al. 2025). The current standard treatment for cardiovascular diseases can modestly slow disease progression (Fordham et al. 2025; Silva et al. 2022). However, they do not directly target the underlying functional decline of cardiomyocytes and offer limited protection against pathological remodeling. There is an urgent need to develop novel therapeutic strategies that address the core pathological mechanisms of cardiac aging.

The quality control and functional homeostasis of mitochondria in cardiomyocytes are crucial for maintaining cardiac energy supply. Mitochondrial homeostasis can be disrupted by multiple mechanisms, including excessive reactive oxygen species (ROS) production, dysregulation of calcium ion balance, and impaired mitophagy and so on (Nguyen et al. 2019; Ravindran and Gustafsson 2025). Mitophagy serves as a central quality control pathway responsible for clearing damaged mitochondria and maintaining the health of the mitochondrial network (Lin, Chen, et al. 2024). If mitophagy initiation or mitophagy flux is congested, the dysfunctional mitochondria are accumulated, thereby hindering energy production and exacerbating oxidative stress, ultimately accelerating the senescent process of heart (Xu et al. 2025).

BCL2‐interacting protein 3 (BNIP3), an atypical Bcl‐2 family protein located in the mitochondrial outer membrane, functions not only as a classical pro‐apoptotic factor but also as a key regulator of mitophagy. Under stress conditions, upregulation of BNIP3 induces mitochondrial depolarization and directly recruits the autophagosome marker protein LC3B via its LC3‐interacting region (LIR), thereby initiating mitophagy (Lu et al. 2023). Sustained or excessive activation of BNIP3 may lead to mitophagy flux aberrant (Y. Chen et al. 2023). In cardiovascular system, BNIP3 has been implicated in various pathological processes, including myocardial ischemia reperfusion injury and heart failure (Q. Q. Wu et al. 2022; Zhang et al. 2022). However, the role of BNIP3 in the aging heart is not yet clear.

Studies have established BNIP3 as a core target gene of the hypoxia‐inducible factor (HIF) family. Its expression is directly regulated by HIF and plays a pivotal role in HIF‐mediated adaptive responses to hypoxia (Huang et al. 2025; Shao et al. 2019). Hypoxia inducible factor‐3α (HIF‐3α) is a member of the HIF family. Traditionally, it has been regarded as a transcriptional repressor mediated by specific protein isoforms, and accumulated in cells under certain hypoxic conditions due to its increased stability (Augstein et al. 2011; Heikkila et al. 2011). Recent research has broadened the understanding of its functions, revealing that beyond its classical repressive role, HIF‐3α also possesses transcriptional activation capabilities and participates in gene expression (Jiang et al. 2024; Tolonen et al. 2020).

The natural aging model is difficult to conduct large‐scale research due to its long duration, high mortality rate, and poor prognosis (Toniolo et al. 2023). A widely used broad‐spectrum anthracycline chemotherapeutic agent (Hyun et al. 2019; Tang and McGoron 2009), doxorubicin induces cardiac damage that closely recapitulates the pathological hallmarks of natural cardiac aging, including mitochondrial dysfunction, cardiomyocyte hypertrophy, and contractile dysfunction (Renu et al. 2018; Lódi et al. 2019; Rawat et al. 2021). This makes doxorubicin a commonly adopted model for investigating myocardial aging.

Recent studies have demonstrated that transplantation of functionally intact mitochondria into damaged cells can effectively improve cellular function and promote tissue repair (Bechet et al. 2025; McCully et al. 2026). However, current research largely relies on mitochondria derived from autologous tissues, whose limited availability and complex preparation severely hinder the clinical translation and application. Mesenchymal stem cells (MSCs), with their capacity for self‐renewal, multi‐lineage differentiation, and potent paracrine activity, represent an ideal cellular source in regenerative medicine (Zhou et al. 2021). Emerging evidence indicates that mitochondrial transfer is an important mechanism through which MSCs exert their therapeutic effects (Lin, Im, et al. 2024). Moreover, the common developmental origin from mesodermal mesenchymal cells suggests an inherent biological affinity of both mesenchymal stem cells and cardiomyocytes (Karp and Leng Teo 2009; Pittenger and Martin 2004). Therefore, we propose to investigate the potential of mitochondria derived from murine bone marrow‐derived mesenchymal stem cells in delaying cardiac aging. We have previously shown that exogenous mitochondria are taken up and retained by cardiomyocytes in the heart, based on time‐lapse imaging of internalization and PCR amplification of human mtDNA in recipient myocardium (Jin et al. 2024). Nevertheless, whether such mitochondrial uptake translates into functional recovery of energy metabolism in the aging heart remains unanswered. Here, we address this gap by combining in vivo fluorescent tracing, ex vivo cardiac imaging, and ATP measurements.

In this study, we established a murine model to mimic clinical phenotypes of the aging heart, and intervened with MSCs derived mitochondria. Our findings demonstrate that during cardiac aging, aberrant upregulation of the transcription factor HIF‐3α leads to overexpression of the mitophagy receptor BNIP3, resulting in mitophagy flux congestion and accelerated cardiomyocyte senescence. Importantly, mitochondrial transplantation effectively reversed the abnormal activation of the HIF‐3α‐BNIP3 axis and restored mitophagy flux congestion, thereby alleviating cardiac aging.

## 2 Materials and Methods

### 2.1 Animal and Cell Models

Male C57BL/6J mice (6–8 weeks old, Vital River Laboratory Animal Technology Co. Ltd.) were used in this study, with all procedures approved by the Animal Care and Use Committee of Shanxi Medical University. Following acclimatization, mice were randomly assigned to three groups: young control group, doxorubicin‐induced aged group, aged, and mitochondrial transplantation group (aged+mito group). Mice in the aged group received intraperitoneal injections of doxorubicin at 2.5 mg/kg every 3 days for six administrations, with a total cumulative dose of 15 mg/kg. For animals in the aged+mito group, the identical doxorubicin regimen was applied, followed by tail vein delivery of isolated mitochondria at 0.5 mg/kg every 2 days for six injections (cumulative dose = 3 mg/kg). Mice in the young group were administered an equal volume of PBS through matched routes.

HL‐1 cardiomyocytes (ZSGB‐Bio) and BMSCs (iCell Bioscience) were cultured in DMEM/F12 medium supplemented with 10% FBS and 1% penicillin–streptomycin at 37°C, 5% CO_2_, and harvested at 70%–90% confluence. HL‐1 cells were assigned to young, aged, and aged+mito groups. Aged group was induced by treating cells with 1.28 μM doxorubicin for 12 h. In the aged+mito group, doxorubicin was removed after 12 h, and the cells were then co‐cultured with 7.5 μg/mL mitochondria for another 12 h.

For chloroquine (CQ) treatment, 200 μM CQ was applied together with doxorubicin (1.28 μM) for 12 h in the aged+CQ group, or alone for 12 h in the young+CQ group. Cells were then processed for immunofluorescence analysis of LC3B and mitochondria.

### 2.2 Echocardiography

Cardiac left ventricular function in mice was assessed using echocardiography. A VisualSonics Vevo 3100 high‐resolution imaging system (Fujifilm, Canada) equipped with an MX‐400 transducer (22–55 MHz) was used. Mice were anesthetized with 2.0% isoflurane for induction, and anesthesia was maintained with 1.0% isoflurane to keep the heart rate between 410 and 450 beats per minute.

### 2.3 In Vivo Fluorescence Imaging and Label‐Free Live‐Cell Imaging

In vivo fluorescence imaging was performed using an IVIS Spectrum system (PerkinElmer, USA). Mice were treated with depilatory cream on the abdominal and thoracic areas the evening prior to the experiment and fasted overnight. On the following day, MitoTracker Red‐labeled mitochondria were administered via tail vein injection. Whole‐body fluorescence imaging was performed at 1, 2, 3, 5, 8, 10, 12, and 24 h post‐injection. Fluorescence signals in the cardiac region were monitored and recorded at each time point.

HL‐1 cells were cultured in imaging‐compatible cell culture dishes (NEST, 801010) and placed into the system (ZIRCON, SC3000, China). Unlabeled isolated mitochondria were added to the culture medium. Time‐lapse images were acquired at designated intervals (every 5 min for 24 h) to monitor the dynamic entry and distribution of exogenous mitochondria within the cells.

### 2.4 WGA Staining

For OCT‐embedded frozen tissue sections, slides were incubated with 20 μg/mL WGA working solution at 37°C for 30 min in the dark. After washing with PBS, slides were mounted with ProLong Gold antifade reagent with DAPI (Invitrogen, P36931). Fluorescence images were captured using a laser scanning confocal microscope (Olympus, FV3000). The cross‐sectional area of cardiomyocytes outlined by WGA was calculated using ImageJ software.

### 2.5 Western Blot

Proteins were resolved by SDS‐PAGE, transferred to PVDF membranes, and blotted with primary and secondary antibodies. Signals were acquired on a Tanon 5200 system and quantified by ImageJ. Normalization was performed using GAPDH/β‐actin (cellular/tissue) or VDAC1 (mitochondria). Antibodies used are listed in Table S1.

### 2.6 SA‐β‐Gal Staining

Cellular and tissue senescence was assessed via senescence‐associated β‐galactosidase (SA‐β‐Gal) staining using a commercial kit (Beyotime, C0602). For OCT‐embedded myocardial frozen sections, samples were fixed in 2% paraformaldehyde/0.2% glutaraldehyde for 30 min at room temperature, then incubated in pH 6.0 staining working solution at 37°C. For HL‐1 cells, adherent cells were fixed for 5 min and stained overnight at 37°C in pH 6.0 working solution. All images were acquired with a Nikon Ti2‐U inverted microscope, and SA‐β‐Gal positivity (area percentage for tissues, cell percentage for cells) was quantified using ImageJ.

### 2.7 Transmission Electron Microscopy

Transmission electron microscopy examined cardiac tissues to assess ultrastructural features. Samples were fixed in 2.5% glutaraldehyde at pH 7.4 and 4°C for 2 h. After washing, post‐fixation used 1% osmium tetroxide for another 2 h. Dehydration proceeded through a graded ethanol series. Specimens were embedded in epoxy resin and polymerized at room temperature for 4 h. Heart tissues were sliced into 70 nm. Images were captured with a transmission electron microscope (Hitachi HT7800).

### 2.8 Isolation of Mitochondria

Mitochondria were isolated from BMSCs by using the Cell Mitochondria Isolation Kit (Beyotime, C3601) with minor modifications. BMSCs were collected by centrifugation at 300 *g* for 5 min. The cell pellet was washed with pre‐cooled PBS and centrifuged again at 300 *g* for 5 min. After discarding the supernatant, the pellet was homogenized and resuspended in mitochondrial isolation reagent containing 1 mM PMSF, followed by incubation on ice for 30 min. The homogenate was centrifuged at 600 *g* for 10 min at 4°C. The supernatant was collected and centrifuged at 12,000 *g* for 15 min at 4°C. The final mitochondrial pellet was resuspended in PBS for subsequent experiments. Isolated mitochondria were quantified using the BCA protein assay (Epizyme Biotech, ZJ102) and resuspended in PBS. The characteristics of isolated mitochondria have been thoroughly characterized in our previous publication (Jin et al. 2024). Herein, we further re‐evaluated the purity and structural integrity of mitochondria isolated for the present study (Figure S1G).

### 2.9 Immunofluorescence Assays

Cells on coverslips (NEST, 801010) were fixed, permeabilized, blocked, and incubated with primary/secondary antibodies, then mounted with DAPI‐containing ProLong Gold antifade reagent (Invitrogen, P36931). Confocal images were captured with an Olympus FV3000 system. Fluorescence intensity and colocalization (Pearson's correlation) were analyzed using ImageJ. Antibodies are detailed in Table S1.

Live‐cell fluorescent staining. For mitochondria, cells were incubated with 125 nM PK Mito Deep Red (Genvivo, PKMDR‐2) or 100 nM MitoTracker Red CMXRos (Invitrogen, M7512) in serum‐free DMEM at 37°C, 5% CO_2_ for 20 min in the dark, washed with PBS, and replenished with complete medium. For lysosomes, cells were stained with 1 μM LysoTracker Green DND‐26 (YEASON, 40738ES50) in serum‐free DMEM under identical conditions for 15 min, followed by PBS washing and medium replacement. Live‐cell images were acquired immediately using an Olympus FV3000 confocal laser scanning microscope.

### 2.10 Mitochondrial Superoxide Levels

Mitochondrial superoxide levels in HL‐1 cells were assessed using the MitoSOX Red mitochondrial superoxide indicator (ThermoFisher, M36007). Cells were incubated with a 500 nM working solution of MSR reagent at 37°C for 20 min. Cells were collected and analyzed immediately by flow cytometry (BD Aria III).

### 2.11 Measurements of ATP Levels

Intracellular ATP levels were measured using the ATP‐Red‐1 assay kit (MCE, HY‐U00451). HL‐1 cells were seeded into opaque‐walled 96‐well plates and subjected to respective treatments. Cells were incubated in 2.5 μM ATP‐Red‐1 reagent at 37°C for 20 min. Fluorescence was measured using a microplate reader (Molecular Devices, SpectraMax iD3) at excitation/emission wavelengths of 510/590 nm.

### 2.12 Bulk RNA Sequencing

Total RNA was extracted from young, aged, aged+mito mouse cardiac tissues, and transcriptomic sequencing was performed using an Illumina NovaSeq platform. The operation was carried out at Sangon Biotech (Shanghai) Co. Ltd.

Functional enrichment analysis of DEGs was conducted using the Kyoto Encyclopedia of Genes and Genomes (KEGG) database, and pathway classification was visualized to annotate biological functions. DEGs were overlapped with a mitochondrial gene set (Mouse Mito Carta 3.0) or the Animal TFDB 3.0 mouse TF list, and the intersection was visualized via a Venn diagram. A heatmap was used to show the expression profile of overlapping genes. Gene Set Enrichment Analysis (GSEA) was conducted to explore the enrichment of cardiac aging‐related gene sets. Transcriptomic data were ranked by the signal‐to‐noise ratio, and enrichment plots were generated to visualize the enrichment score (ES), normalized enrichment score (NES), and statistical significance (*p* value).

### 2.13 Proteomic Sequencing and Differential Analysis

Total proteins were extracted from young and aged HL‐1 cells, and label‐free quantitative proteomics was performed using nano liquid chromatography coupled with tandem mass spectrometry (nanoLC‐MS/MS). Raw data were processed with DIANN software (1.8.1). The operation was carried out at Biotree Biotech Co. Ltd.

Differentially expressed proteins (DEPs) between young and aged groups were analyzed and visualized by volcano plot. DEPs were defined as proteins with |log_2_FC| ≥ 1 and *p* < 0.05: up‐regulated proteins (red dots) with log_2_FC > 1, down‐regulated proteins (blue dots) with log_2_FC < −1. Statistical analysis was performed using Student's *t*‐test, and the volcano plot was generated with the ggplot2 package in R software. *p* < 0.05 was considered statistically significant.

### 2.14 RT‐qPCR

Total RNA was extracted from cardiac tissue or HL‐1 cells with Trizol reagent (YALEPIC, YR23014). cDNA synthesis was conducted using TransScript Uni All‐in‐One First‐Strand cDNA Synthesis SuperMix for qPCR (TransGen, AU341‐02). qPCR of target mRNA was performed with M5 HiPer Realtime PCR Super mix with Low Rox (Mei5 Biotech, MF797) on a QuantStudio 3 Real‐Time PCR instrument. Cycle threshold (Ct) values were recorded. Relative gene expression was calculated using the 2^(−ΔΔCt)^ method, normalized to β‐actin. All primer sequences are listed in Table S2.

### 2.15 Plasmids Transfection and siRNA Assays

Plasmids were obtained from GeneChem Co. Ltd. (Shanghai, China). HL‐1 cells were transfected with 2.5 μg plasmid DNA using Lipo8000 (Beyotime, C0533) at 70% confluence according to the manufacturer's protocol. After 24 h, cells were harvested, and overexpression efficiency was verified by Western blotting and qPCR.

siRNA transfection was performed using siRNA‐mate plus (GenePharma, G04026) in 24‐well plates at a final concentration of 20 nM. Cells were incubated for 48 h before harvesting for qPCR and Western blot analysis. The siRNA sequence for Hif‐3α was 5′‐CCAUGGAUGAUGACUUCCATT‐3′.

### 2.16 Chromatin Immunoprecipitation (Chip) Assay

The Chip Assay Kit (CST, 9005) was employed following the manufacturer's instructions. Briefly, cells were first transfected with plasmids carrying an HA tag (pcDNA3.1‐HA and pcDNA3.1‐Hif‐3α‐HA) for 24 h. After cross‐linking, chromatin was fragmented by sonication (30 s ON, 30 s OFF, 10 cycles) to lengths between 150 bp and 900 bp. Immunoprecipitation was then carried out using either HA‐Tag antibody (CST, 3724; 1: 50) or an equal amount of IgG antibody, with IgG serving as the negative control. The DNA recovered from the Chip procedure was eluted, reversed from cross‐linking, and purified before being measured by RT‐qPCR. Results are presented as the percentage of the relate Input sample. The primer sequences used for Chip‐PCR are provided in Table S3.

### 2.17 Single‐Cell RNA‐Seq Data Acquisition and Analysis

Human heart single‐cell RNA‐seq datasets were obtained from the GEO database (GSE156703) and the Heart Cell Atlas. Data quality control, normalization, and integration were performed using the Seurat package (v4.4.1). Dimensionality reduction and clustering were conducted via the UMAP algorithm, and cell clusters were annotated based on canonical cell type‐specific markers. The expression matrices of target genes (BNIP3, HIF3A) were extracted, and gene expression visualization under UMAP projection was realized by FeaturePlot. For the cardiomyocyte subset, violin plots were generated using VlnPlot to quantify gene expression differences between groups. Statistical analysis was performed using the Wilcoxon rank‐sum test, and *p* < 0.0001 was defined as extremely significant difference.

### 2.18 Data Analysis

All images were analyzed and quantified using ImageJ software. Data are presented as mean ± standard deviation (SD), with *n* = 3−11 independent experiments. Statistical analysis was performed using GraphPad Prism 9 software. Statistical analyses were performed using GraphPad Prism software. For comparisons between two groups, an unpaired two‐tailed Student's *t*‐test was used. For comparisons involving three or more groups, one‐way analysis of variance (ANOVA) followed by Tukey's multiple comparisons post hoc test was applied. A *p*‐value of less than 0.05 was considered statistically significant.

## 3 Results

### 3.1 Doxorubicin Triggers the Senescence Program in Cardiomyocytes

**FIGURE 1:**
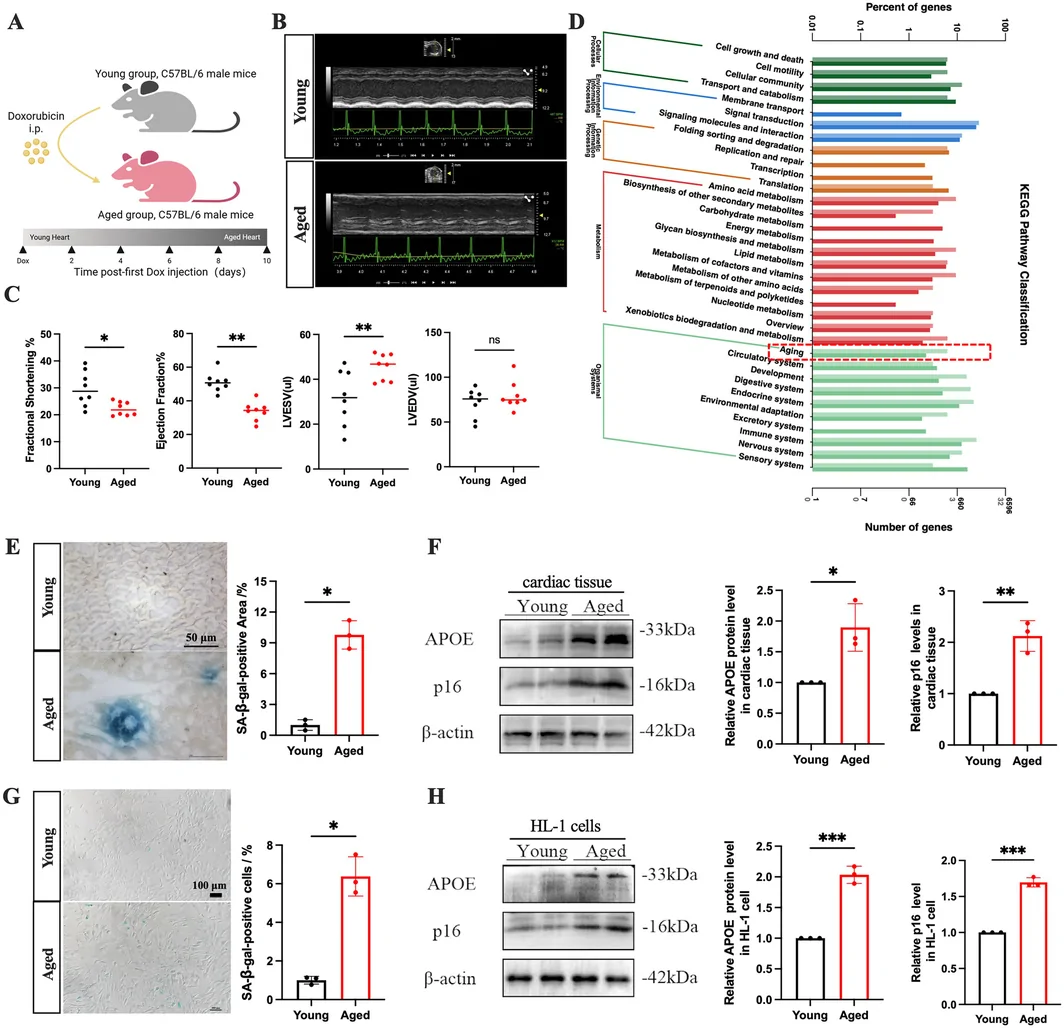
Establishment and characterization of the murine model of aging heart. (A) Schematic showing the study design of DOX‐induced cardiac aging in animals. (B) Representative echocardiographic images of cardiac structure and function. (C) Calculations and quantifications of FS, EF, LVESV, and LVESD in each group (*n* = 8 per group). (D) KEGG analysis for the DEGs. X‐axis indicated the enrichment score (−log_10_ [*p* value]) of DEGs. Y‐axis showed the names of enriched pathways. Node size represented the number of enriched differential genes. The *p*‐value was represented by a color scale. (E) SA‐β‐gal staining was performed on frozen sections of cardiac tissues from young and aged groups, and the corresponding quantitative analysis is shown on the right. Scale bars, 50 μm. (F) Expression and quantification of senescence‐associated proteins (APOE and p16) in cardiac tissue by Western blot analysis. (G) SA‐β‐Gal staining of HL‐1 cells, with corresponding quantitative analysis shown on the right (*n* = 3 per group). Scale bars, 100 μm. (H) Expression and quantification of senescence‐associated proteins (APOE and p16) in HL‐1 cells by Western blot analysis. Bars represent mean ± SD. *p < 0.05, **p < 0.01, ***p < 0.001 Statistical significance was assessed using an unpaired two‐tailed Student's *t*‐test.

The establishment of the mouse cardiac aging model is shown in Figure 1A. Echocardiographic assessment revealed that doxorubicin treatment significantly reduced left ventricular fractional shortening (FS) and ejection fraction (EF), and increased left ventricular end‐systolic volume (LVESV), whereas left ventricular end‐diastolic volume (LVEDV) remained unchanged (Figure 1B,C). These results indicate that doxorubicin induced cardiac overload and impaired systolic function in mice (Rawat et al. 2021; Renu et al. 2018). Transcriptomic analysis of cardiac tissue suggested potential aging‐related mechanisms. The KEGG pathway enrichment analysis indicated that the pathways related to aging were significantly enriched (Figure 1D). This provides bioinformatic evidence that doxorubicin induces cardiac aging and physiological functional decline.

Then we analyzed the senescent phenotype in cardiac tissue. Compared with the control group, the positive area of the senescence‐associated β‐galactosidase (SA‐β‐Gal) in the aged group significantly increased (Figure 1E). Consistently, the expression levels of the senescence marker proteins apolipoprotein E (APOE) and p16^INK4a^ (p16) were also upregulated (Figure 1F). Together, these results demonstrate that doxorubicin successfully induces cardiac aging in vivo.

To isolate the direct effect of cardiomyocyte senescence from the complexities of the in vivo environment, we established an in vitro model using senescent HL‐1 cells. After treating HL‐1 cells with doxorubicin for 12 h, we detected the same senescent biomarkers: enhanced SA‐β‐Gal activity and upregulated expression of APOE and p16 (Figure 1G,H). The high concordance between in vivo and in vitro results provides strong evidence that doxorubicin directly activates the cardiomyocyte senescence program.

### 3.2 Mitophagy Flux Congestion Contributes to Mitochondrial Dysfunction in the Aging Heart

**FIGURE 2:**
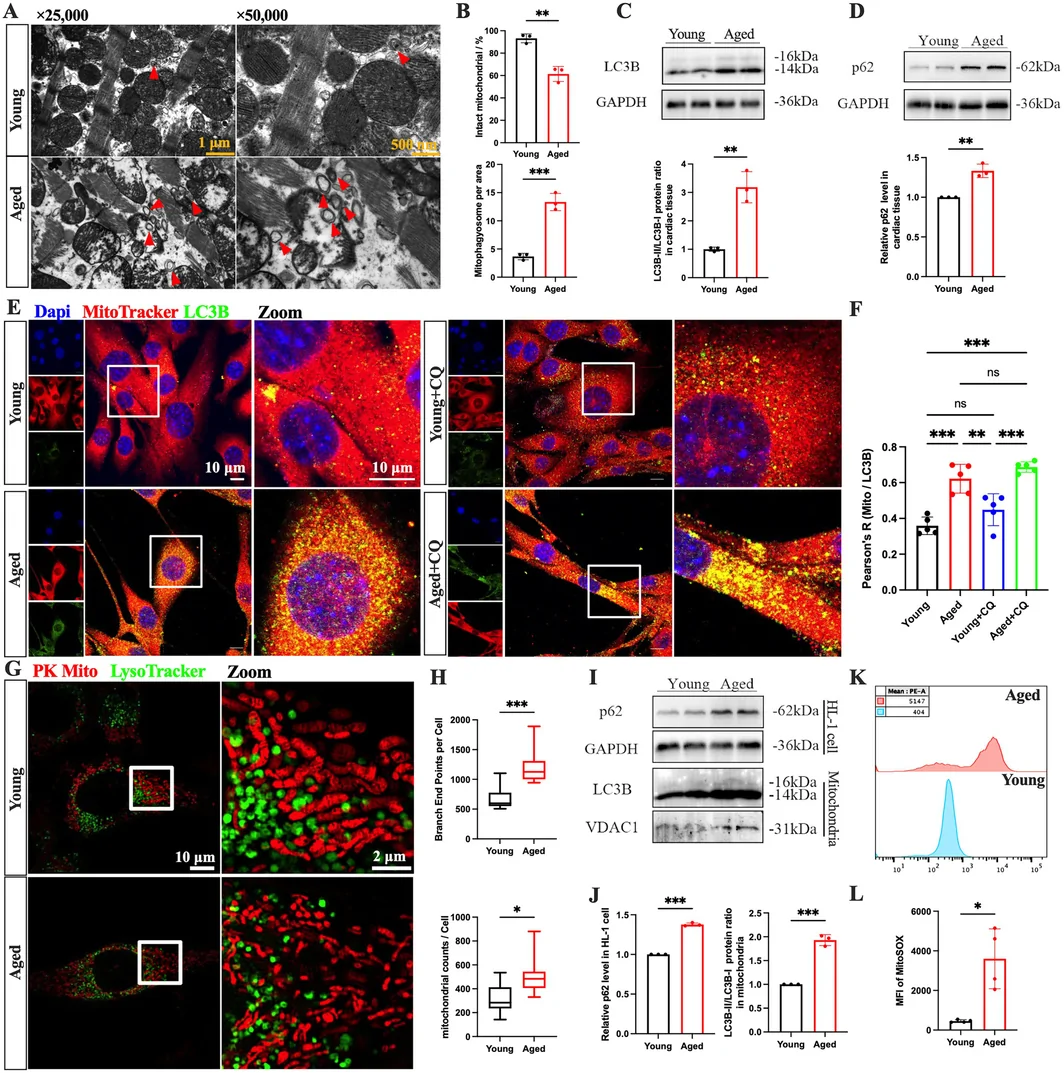
Mitophagy flux congestion and mitochondrial dysfunction in the aging heart. (A) Representative TEM images of myocardial tissue. Left panels: Scale bar = 1 μm (× 25,000); right panels: Scale bar = 500 nm (× 50,000). Red arrows indicate mitophagosomes. (B) Quantification of Mitophagosome numbers per area and the ratio of intact mitochondria (*n* = 3 per group). (C) Expression and quantification of LC3B in cardiac tissues by Western blot analysis (*n* = 3 per group). (D) Expression and quantification of p62 in cardiac tissues by Western blot analysis (*n* = 3 per group). (E) Representative fluorescence images of mitochondria (red) and LC3B (green) in HL‐1 cells. Zoomed panels (right) correspond to the boxed areas in the merged images. Scale bars, 10 μm (main and zoomed panels). (F) Quantification analysis of the colocalization percentage between mitochondria and LC3B (*n* = 5 per group). (G) Representative live‐cell SIM images showing mitochondria (PK Mito, red) and lysosomes (Lyso Tracker, green) in young and aged groups. Zoomed panels (right) correspond to the boxed areas in the merged images. Scale bars: 10 μm (main); 2 μm (zoomed). (H) Quantification of mitochondrial parameters (branch end points and number per cell) (*n* = 8 per group). (I, J) Expression and Quantification of autophagy‐related proteins in young and aged HL‐1 cells: P62 in cell lysate and LC3B‐II/I in mitochondrial components, analyzed by Western blot (*n* = 3 per group). (K, L) Flow cytometry analysis of MitoSOX levels in the Young and senescent HL‐1 cells, with statistical data shown on the bottom (*n* = 3 per group). Bars represent mean ± SD. *p < 0.05, **p < 0.01, ***p < 0.001 Statistical significance was assessed using an unpaired two‐tailed Student's *t*‐test.

Studies have shown mitochondrial dysfunction accompanies cardiac aging (Q. Chen et al. 2020; Wang et al. 2022; Xu et al. 2025), we further investigated the state of mitochondria during cardiac aging. Transmission electron microscopy (TEM) revealed significantly more mitophagosomes in the aging heart (Figure 2A,B). To determine whether this accumulation reflects enhanced autophagic initiation or impaired autophagic degradation, we performed multi‐level validation.

Then we assessed LC3B and p62 levels in aging cardiac tissue and observed that p62 was elevated along with LC3B‐II (Figure 2C,D). Consistent with these results in vivo, we observed similar increases in p62 and mitochondrial‐associated LC3B‐II in senescent HL‐1 cells (Figure 2I,J). Immunofluorescence staining showed that the number of LC3B puncta markedly increased in senescent HL‐1 cells and enhanced their colocalization with mitochondria, and chloroquine treatment led to a marked increase in colocalization in young HL‐1 cells, but failed to produce a further increase in aged HL‐1 cells, indicating that the degradative arm of mitophagy is already functionally saturated in senescent cardiomyocytes (Figure 2E,F). Together with the concurrent elevation of LC3B and p62 proteins in aged cells, these findings indicate that mitophagy initiation is enhanced while downstream degradation is impaired, revealing a state of mitophagy flux blockade in senescent cardiomyocytes. Moreover, no changes in the localization of mitochondria and lysosomes were observed (Figure 2G), indicating that the damaged mitochondria were encapsulated within autophagosomes but were not effectively cleared. Collectively, our data indicate that increased mitophagosome formation in senescent HL‐1 cells, together with unaltered downstream degradation function, leads to congestion of the mitophagy flux.

Live cell imaging revealed that, compared to healthy cardiomyocytes, senescent cardiomyocytes exhibited shorter, more rounded mitochondria with increased mitochondrial fragmentation (Figure 2G,H). We further assessed mitochondrial functional status. The senescent HL‐1 cells showed a significant increase in mitochondrial superoxide (MitoSOX) levels (Figure 2K,L). These results indicate that mitophagy flux congestion in senescent cardiomyocytes leads to the accumulation of dysfunctional mitochondria, which exacerbates oxidative stress and disrupts cardiac function, ultimately accelerating heart aging.

### 3.3 Mitochondrial Transplantation Rejuvenates Aging Heart and Alleviates Cardiomyocyte Senescence

**FIGURE 3:**
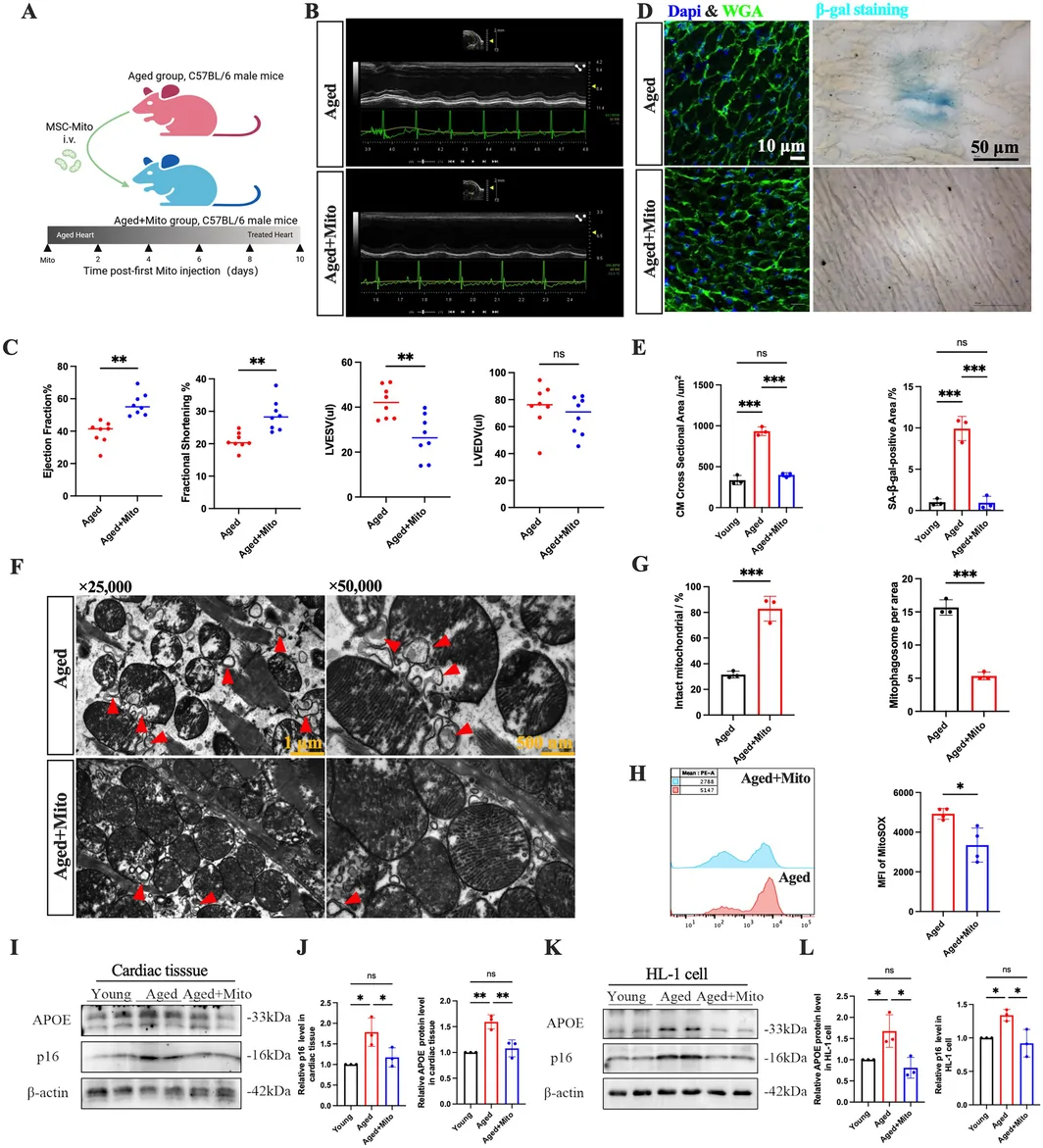
Mitochondrial transplantation rejuvenates aging heart in mice. (A) Schematic showing the study design for BMSC‐derived mitochondrial transplantation therapy in aging heart. (B) Representative echocardiographic images of cardiac structure and function. (C) Calculations and quantifications of FS, EF, LVESV and LVESD in each group. (D) Representative results of WGA and SA‐β‐gal staining on frozen sections of cardiac tissues are presented. Scale bars, 10 μm (WGA staining) and 50 μm (SA‐β‐gal staining). (E) Statistical analysis of cardiomyocyte cross‐sectional area per μm^2^ and the percentage of SA‐β‐gal‐positive area in frozen heart sections from aged and aged+mito groups (*n* = 3 per group). (F) Representative TEM images of cardiac tissue. Left panels: Scale bar = 1 μm (× 25,000); right panels: Scale bar = 500 nm (× 50,000). Red arrows indicate mitophagosome. (G) Quantification of Mitophagosomes number per area and the ratio of intact mitochondria. (*n* = 3 per group). (H) Flow cytometry analysis of MitoSOX levels in the Aged and aged+mito HL‐1 cells. Statistical analysis of these data is provided on the right. (*n* = 3 per group). (I, J) Expression and quantification of senescence‐associated proteins (APOE and p16) in myocardia by Western blot (*n* = 3 per group). (K, L) Expression and quantification of senescence‐associated proteins (APOE and p16) in HL‐1 cells by Western blot (*n* = 3 per group). Comparisons between two groups were performed using the unpaired *t*‐test. For comparisons among multiple groups, one‐way analysis of variance (ANOVA) was used. Bars represent mean ± SD. *p < 0.05, **p < 0.01, ***p < 0.001.

The above findings indicate that the process of autophagy in mitochondria is disrupted, leading to mitochondrial dysfunction during cardiac aging. We attempted to alleviate this process by transplanting MSCs derived mitochondria, according to the scheme outlined in Figure 3A. Images and videos showing the transplantation of mitochondria into cardiac tissue and HL‐1 cardiomyocytes can be found in Figure S1 and Movie S1. Transplantation of MSCs‐derived mitochondria effectively improved cardiac function in mice. Echocardiography showed that compared with the aged group, the aged+mito group exhibited significant recovery in left ventricular fractional shortening (FS), ejection fraction (EF), and left ventricular end‐systolic volume (LVESV) (Figure 3B,C). WGA staining revealed attenuated cardiomyocyte hypertrophy after transplantation, and SA‐β‐Gal staining demonstrated a marked reduction in positive areas (Figure 3D,E).

TEM observations revealed that mitochondrial transplantation reduced the abnormal accumulation of mitophagosomes and restored the number of intact mitochondria as well as cristae density (Figure 3F,G). Flow cytometry also showed a significant decrease in MitoSOX levels in the aged+mito group (Figure 3H), indicating mitochondrial oxidative stress damage was alleviated. Then we examined senescence biomarkers in the cardiac tissue and HL‐1 cells. Western blot analysis demonstrated that the expression of APOE and p16 was significantly reduced following transplantation (Figure 3I,L). Taken together, these results indicate that mitochondrial transplantation could restore mitochondrial function and alleviate cardiac aging.

### 3.4 BNIP3 Is the Key Mediator of Aging Heart and Mitophagy Flux Congestion

**FIGURE 4:**
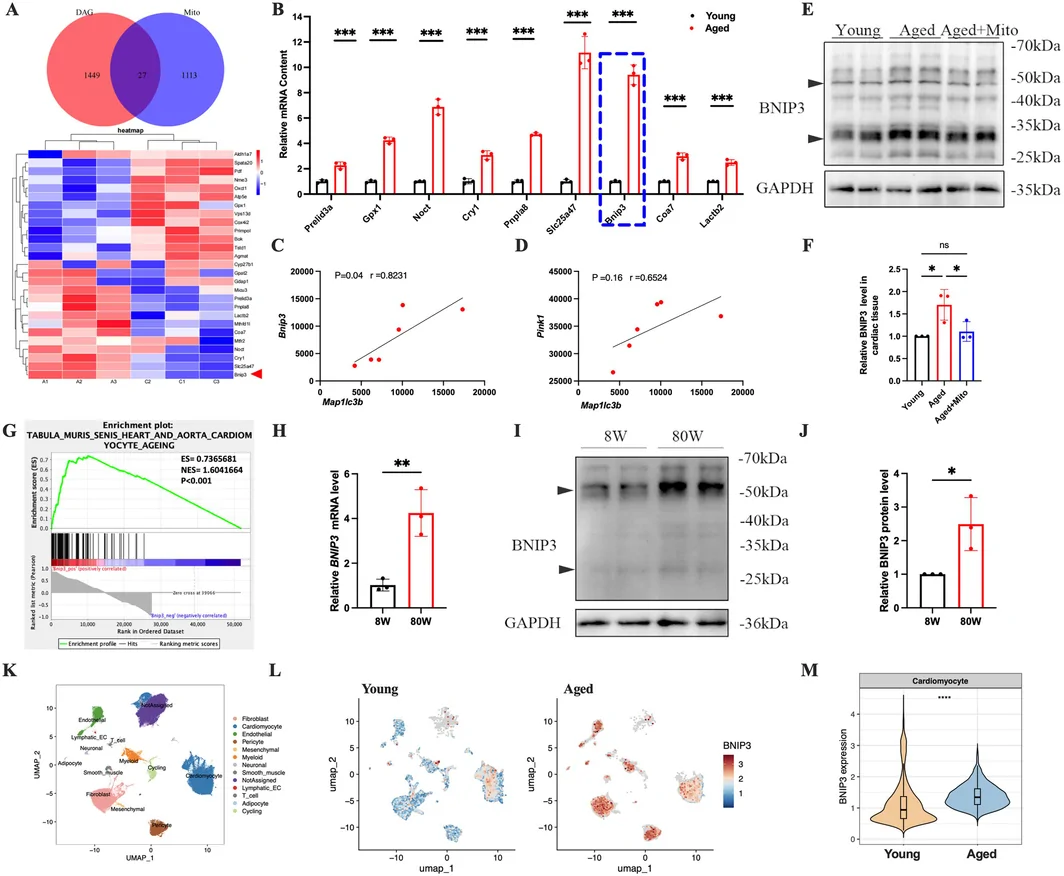
Multi‐omics screening identifies senescence associated mitophagy genes. (A) Analysis of mitochondrial‐related DEmRNAs in young and aged groups. The expression values were represented by a color scale. A1, A2, A3: Aged tissue, C1, C2, C3: Young tissue. (B) Analysis of mRNA profiling for differentially expressed genes in young versus aged groups (*n* = 3 per group). (C, D) The correlation analysis between LC3B (*Map1lc3b*) and *Bnip3/Pink1*. (E, F) Expression and quantification of BNIP3 protein in doxorubicin‐induced senescent myocardium by Western blot (*n* = 3 per group). (G) GSEA on the “TABULA_MURIS_SENIS_HEART_AND_AORTA_CARDIOMYOCYTE_AGEING” gene set, with genes ranked by Pearson correlation to *Bnip3*. (H) Statistical analysis of *BNIP3* mRNA expression in the cardiac tissues of naturally aged mice (*n* = 3 per group). (I, J) Expression and quantification of BNIP3 protein in the cardiac tissues of naturally aged mice by Western blot (*n* = 3 per group). (K) Cell type annotation of single‐cell transcriptomes from human heart tissues was performed via UMAP dimensionality reduction and clustering. Datasets were from the GSE156703 and Heart_Cell_Atlas datasets (https://www.heartcellatlas.org/v1.html). (L) UMAP gene expression visualization showed the expression of BNIP3. (M) Violin plot quantitative analysis of the expression levels of BNIP3 in cardiomyocytes. Comparisons between two groups were performed using the unpaired *t*‐test. For comparisons among multiple groups, one‐way analysis of variance (ANOVA) was used. Bars represent mean ± SD. *p < 0.05, **p < 0.01, ***p < 0.001.

To further investigate the relationship between mitophagy flux and cardiac aging, as well as the mechanism by which mitochondrial transplantation alleviates aging, we performed RNA‐seq on cardiac tissues. By intersecting the differentially expressed genes (DEGs) from RNA‐seq between the young group and the aged group with mitochondrial related genes, we identified 27 mitochondrial associated DEGs (Figure 4A), including 14 upregulated and 13 downregulated genes. Nine of these upregulated genes were validated by PCR (Figure 4B). Notably, the mitophagy related gene *Bnip3* was significantly upregulated in the aged group. Correlation analysis revealed a significant positive relationship between *Bnip3* and the autophagy marker *Map1lc3b* expression levels (Figure 4C), suggesting that BNIP3 may play a key role in the mitophagy flux congestion of the senescent process. Protein analysis confirmed that BNIP3 expression was markedly high in the aged group compared with the young group (Figure S2A,B). These findings indicate that abnormal accumulation of BNIP3 may be a critical event leading to subsequent mitophagy flux congesting.

We also examined the canonical mitophagy pathway mediated by PINK1/Parkin. In vivo and in vitro experiments demonstrated that Parkin protein levels remained consistent between aged and young groups (Figure S2C,D). Furthermore, the correlation between *Map1lc3b* and *Pink1* (*r* = 0.6524) was significantly weaker than the correlations of *Map1lc3b* with *Bnip3* (*r* = 0.8231) (Figure 4D). These data collectively indicate that the mitophagy flux congestion in the aging heart is primarily mediated by upregulation of the selective receptor BNIP3, rather than through the canonical PINK1/Parkin pathway.

Then we conducted RNA sequencing of the aged group and aged+mito group and found that *Bnip3* was significantly downregulated after treatment (Figure S2E). Subsequent qPCR and Western blot analyses confirmed that mitochondrial transplantation effectively reversed the upregulation of BNIP3 induced by the senescent process (Figure 4E,F, Figure S2F). Gene set enrichment analysis (GSEA) performed on the expression matrices of the young group and aged group revealed that the gene set “TABULA_MURIS_SENIS_HEART_AND_AORTA_CARDIOMYOCYTE_AGEING” was significantly enriched in the aged group (ES = 0.772, NES = 1.902, *p* < 0.001) (Figure S2G). Then genes were ranked by their correlation with *Bnip3* expression, followed by GSEA on the expression data from the young group and aged group. The “TABULA_MURIS_SENIS_HEART_AND_AORTA_CARDIOEMYOCYTE_AGEING” gene set showed significant enrichment in the *Bnip3*‐positively correlated genes (ES = 0.737, NES = 1.604, *p* < 0.001) (Figure 4G).

Hearts from naturally aged mice (80‐week‐old) exhibited markedly increased BNIP3 transcript and protein levels compared with young controls (8‐week‐old) (Figure 4H–J), consistent with the BNIP3 upregulation detected in doxorubicin‐triggered cardiac senescence. To examine the expression of BNIP3 in aging heart of human, the GSE156703 and Heart_Cell_Atlas datasets were analyzed. Following cell type annotation of single‐cell transcriptomic data from human heart tissues via UMAP dimensionality reduction and clustering (Figure 4K), gene expression visualization and quantitative analysis were performed. UMAP projection results showed that, compared with the young group, the number of BNIP3 highly expressed cells was significantly increased (Figure 4L), indicating an overall upregulation of BNIP3 transcriptional activation in aging heart. Violin plot quantitative analysis of BNIP3 expression revealed that the expression of BNIP3 in senescent cardiomyocytes was significantly higher than that in the young group (Figure 4M). These human single‐cell sequencing results are highly consistent with the experimental findings obtained in our murine model of cardiac aging, suggesting that the upregulation of BNIP3 may be a key factor in cardiac aging and mitochondrial therapy.

### 3.5 Mitochondrial Transplantation Restores Mitophagy Flux by Downregulating BNIP3 in Aging Heart

**FIGURE 5:**
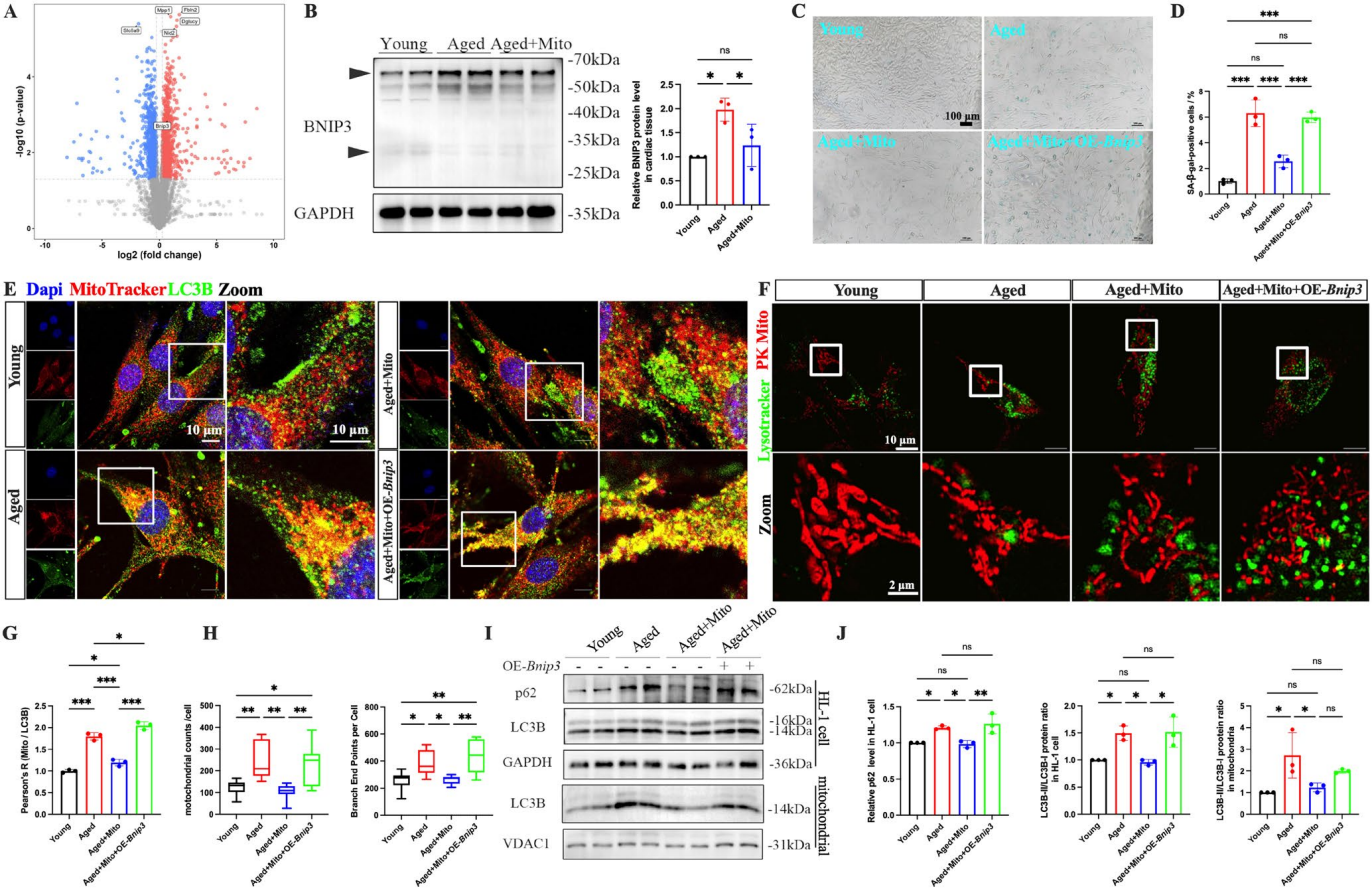
Mitochondrial transplantation downregulates BNIP3 and restores mitophagy flux in aging heart. (A) Volcano plot of proteomic sequencing for the young and old groups of HL‐1 cells. (B) Expression of BNIP3 protein in HL‐1 cell by Western blot analysis, with corresponding quantitative analysis shown on the right (*n* = 3 per group). (C) Representative images of SA‐β‐Gal staining in HL‐1 cells, Scale bars, 100 μm. (D) Quantitative analysis of SA‐β‐Gal staining in HL‐1 cells (*n* = 3 per group). (E) Representative fluorescence images of mitochondria and LC3B in HL‐1 cells. Zoomed panels (right) correspond to the boxed areas in the merged images. Scale bars, 10 μm (main and zoomed panels). (F) Representative live‐cell SIM images showing mitochondria (PK Mito, red) and lysosomes (Lyso Tracker, green) in young and aged groups. Zoomed panels (right) correspond to the boxed areas in the merged images. Scale bars: 10 μm (main); 2 μm (zoomed). (G) Quantitative analysis of co‐localization of LC3B and mitochondria in HL‐1 cells. (*n* = 3 per group) (H) Quantification of mitochondrial parameters (branch end points and number per cell) (*n* = 8 per group). (I, J) Expression and quantification of autophagy‐related proteins (p62 and LC3B‐II/I) in HL‐1 cells (Young, Aged, Aged+Mito, and BNIP3‐overexpressing Aged+Mito groups, *n* = 3 per group). Comparisons between two groups were performed using the unpaired *t*‐test. For comparisons among multiple groups, one‐way analysis of variance (ANOVA) was used. Bars represent mean ± SD. *p < 0.05, **p < 0.01, ***p < 0.001.

Proteomic volcano plot analysis further verified that BNIP3 expression was significantly upregulated in senescent HL‐1 cells (Figure 5A). To directly validate the role of BNIP3 in the aging heart and mitochondrial therapy, we conducted a series of experiments in senescent HL‐1 cells. Immunofluorescence staining, qPCR, and Western blot analyses consistently demonstrated that mitochondrial transplantation downregulated BNIP3 expression at both mRNA and protein levels (Figure 5B, Figure S3B,C). These findings indicate that mitochondrial transplantation can alleviate the aberrant upregulation of BNIP3 in senescent cardiomyocytes. Immunohistochemical data from the Human Protein Atlas (HPA) showed that BNIP3 protein was positively expressed in human myocardial cells, with cytoplasmic localization (Figure S3A).

To determine whether BNIP3 overactivation alone is sufficient to induce aging‐related phenotypes, we overexpressed BNIP3 in HL‐1 cardiomyocytes (Figure S3D,E). Western blot analysis showed that BNIP3 overexpression significantly upregulated the protein levels of senescence markers p16 and APOE compared to vector‐transfected controls (Figure S3G,H). SA‐β‐Gal staining further revealed a marked increase in the percentage of senescent cells upon BNIP3 overexpression (Figure S3I,J). Moreover, qPCR analysis demonstrated that BNIP3 overexpression concurrently elevated the mRNA levels of both *Map1lc3b* and *Sqstm1* (p62), a pattern indicative of autophagic flux blockade (Figure S3F). Collectively, these results suggest that BNIP3 overactivation alone is sufficient to drive both cellular senescence and autophagic congestion in cardiomyocytes.

To further investigate whether BNIP3 downregulation is essential for the protective effects of mitochondrial transplantation, we performed a functional reversal experiment by overexpressing BNIP3 in the context of mitochondrial transplantation. SA‐β‐Gal staining (Figure 5C,D) demonstrated that forced BNIP3 overexpression abolished the protective benefits of mitochondrial transplantation. The senescent staining intensity in the Aged+Mito+OE‐*Bnip3* group matched that of the aged control group. This result indicated that BNIP3 overexpression abolishes the senescence‐attenuating effect of mitochondrial transplantation. Further immunofluorescence co‐localization analysis revealed that after mitochondrial therapy, the co‐localized area of LC3B with mitochondria was significantly reduced in senescent HL‐1 cells, and was again enhanced in the BNIP3 overexpressing group (Figure 5E,G). In contrast, no significant difference in mitochondrial‐lysosome co‐localization was observed among the groups (Figure 5F). Results showed that BNIP3 overexpression markedly attenuated the restorative effect of mitochondrial transplantation on mitophagy flux, as evidenced by the re‐accumulation of both LC3B‐II and p62 (Figure 5I,J). Simultaneously, we analyzed isolated mitochondrial fractions (Figure 5I,J). The results showed that transplantation significantly reduced the accumulated level of LC3B‐II in the mitochondria of senescent cardiomyocytes, whereas BNIP3 overexpression reversed this effect, leading to a re‐elevation of LC3B‐II. This indicates that aberrant BNIP3 expression directly perturbs the process of mitophagy.

These results demonstrate that mitochondrial transplantation effectively alleviates mitophagy flux congestion in senescent cardiomyocytes, and this protective effect depends on the downregulation of the mitophagy receptor BNIP3. Mitophagy flux congestion leads to the accumulation of dysfunctional mitochondria, which subsequently disrupts mitochondrial network integrity (Figure 5F,H), and accelerates cardiomyocyte senescence (Figure 5C). These findings collectively indicate that BNIP3 serves as a key molecular target through which mitochondrial transplantation rejuvenates cardiomyocyte senescence. The intervention likely alleviates the accumulation of mitophagosomes by downregulating BNIP3 expression, thereby reviving mitophagy flux congestion, and ultimately delaying senescence in cardiomyocytes.

### 3.6 HIF‐3α Regulates BNIP3 Expression and Disrupts Mitophagy in Senescent Cardiomyocytes

**FIGURE 6:**
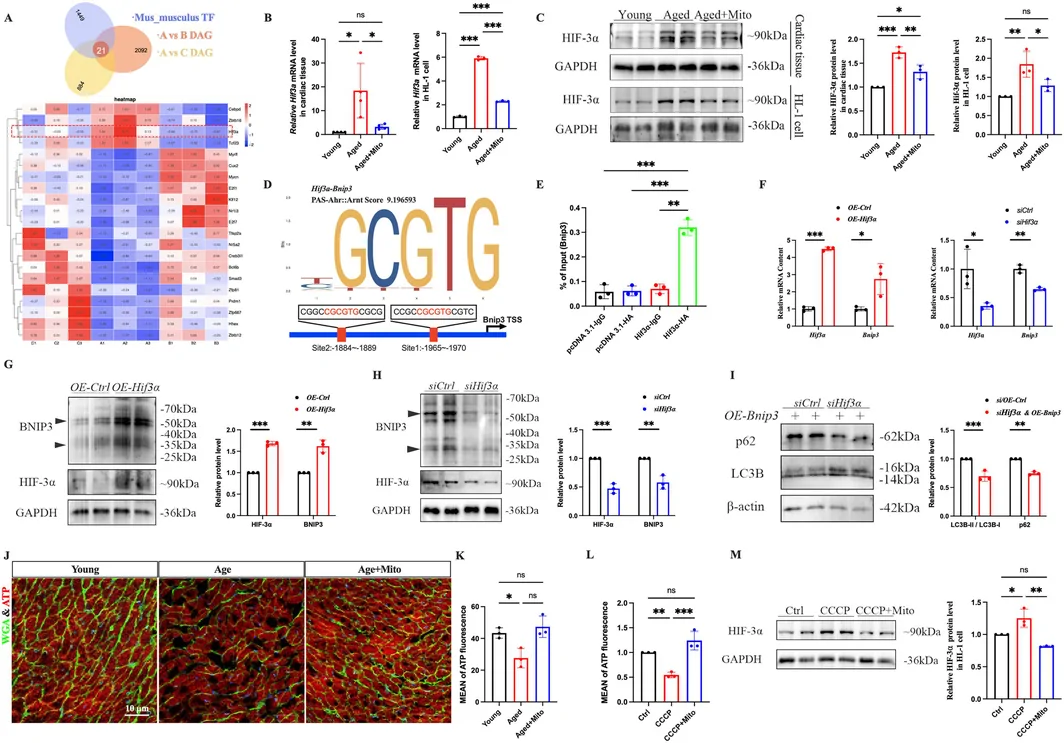
Abnormally upregulated HIF‐3α drives the high expression of BNIP3. (A) Interaction analysis of DEmRNAs and transcriptional factor (TF) subsets. The expression values were represented by a color scale. A1, A2, A3: Aged tissues, B1, B2, B3: Aged+Mito tissues, C1, C2, C3: Young tissues. (B) Statistical analysis of *Hif3α* mRNA expression in cardiac tissue and HL‐1 cells (*n* = 3 per group). (C) Expression and quantification of HIF‐3α protein in cardiac tissue and HL‐1 cells, with corresponding quantitative analysis shown on the right (*n* = 3 per group). (D) JASPAR prediction result shows HIF‐3α shares a specific motif (CGCGTG) with *Bnip3*. (E) Chip‐qPCR results indicated the binding of HIF‐3α on the site 2 of BNIP3 promoter. (F) *Bnip3 and Hif3α* mRNA expression in HL‐1 cells with HIF‐3α overexpression or knockdown. (*n* = 3 per group). (G) Expression and quantification of BNIP3 and HIF‐3α in *Hif3α* overexpressing HL‐1 cells by Western blot analysis (*n* = 3 per group). (H) Expression and quantification of HIF‐3α and BNIP3 in *Hif3α* knockdown HL‐1 cells by Western blot analysis (*n* = 3 per group). (I) Expression and quantification of p62 and LC3B‐II/I in *Hif3α* knockdown and *Bnip3* overexpression cells. (*n* = 3 per group). (J) Representative confocal images of cardiac tissues, scale bar: 10 μm, (red: ATP, green: WGA, blue: DAPI, *n* = 3 per group). (K) Quantitative analysis of ATP levels in cardiac tissues (*n* = 3 per group). (L) Quantitative analysis of ATP levels in the Control, CCCP and CCCP+Mito groups (*n* = 3 per group). (M) Expression and quantification of HIF‐3α in the Control, CCCP and CCCP+Mito groups by Western blot analysis (*n* = 3 per group). Comparisons between two groups were performed using the unpaired *t*‐test. For comparisons among multiple groups, one‐way analysis of variance (ANOVA) was used. Bars represent mean ± SD. *p < 0.05, **p < 0.01, ***p < 0.001.

To elucidate the upstream regulatory mechanism of BNIP3, we screened for differentially expressed transcription factors (TFs) based on transcriptomic data using the Animal TFDB 3.0 mouse TF list, and identified 21 significantly altered TFs (Figure 6A). Through systematic screening, HIF‐3α was suggested as a potential regulator of *Bnip3*, a hypothesis which warrants experimental validation in subsequent studies. We first examined immunohistochemical data from the Human Protein Atlas (HPA), which showed that HIF‐3α protein (sample ID: HPA041141) was expressed in the nucleus of normal human myocardial tissue (Figure S4B). Then we assessed the co‐expression relationship between *HIF‐3α* and *BNIP3* using the GEPIA2 platform, which integrates data from the GTEx project. Analysis of normal human left ventricular tissues revealed a significant positive correlation between their expression levels (Figure S4A). These results suggest a potential regulatory association between HIF‐3α and BNIP3 in human cardiac tissue.

Subsequently, we characterized HIF‐3α expression in doxorubicin‐induced senescent myocardium and HL‐1 cardiomyocytes (Figure 6B,C). HIF‐3α protein abundance was markedly elevated in senescent cardiomyocytes, while mitochondrial transplantation robustly reversed this upregulation. Consistent with observations from the doxorubicin‐induced cardiac aging model, both *Hif3a* mRNA and HIF‐3α protein levels were significantly higher in myocardial tissues isolated from 80‐week‐old naturally aged mice compared with 8‐week‐old young controls (Figure S4E–G). Single‐cell transcriptomic profiling of human cardiac datasets (GSE156703, Heart Cell Atlas) further validated increased HIF3A transcripts in aged human cardiomyocytes (Figure S4C–E). Collectively, these data reveal conserved upregulation of HIF‐3α during physiological cardiac aging across murine and human species.

Then we analyzed and predicted the regulatory relationship between HIF‐3α and *Bnip3* using the JASPAR database. Results showed the transcription factor HIF‐3α has a high affinity binding site (Score: ~9.20) in the promoter region of *Bnip3* (Figure 6D), implying that HIF‐3α may directly govern BNIP3 transcription. We then performed dual‐luciferase reporter assays using the full‐length *Bnip3* promoter fragment containing the predicted binding sequence (Figure S4I). Co‐transfection of HIF‐3α significantly increased reporter luciferase activity, confirming that HIF‐3α is capable of activating transcription driven by the intact *Bnip3* promoter. To further validate the physical interaction between HIF‐3α and the endogenous Bnip3 promoter at the molecular level, we conducted ChIP‐qPCR. The results confirmed robust enrichment of HIF‐3α at Site 2 of the *Bnip3* promoter (Figure 6E), directly verifying this genomic region as the bona fide binding locus for HIF‐3α. To rule out indirect regulatory effects arising from competitive binding with other HIF isoforms, we detected HIF‐1α protein abundance in all experimental groups. In contrast, the expression of HIF‐1α, a principal member of the hypoxia‐inducible factor family (Yao et al. 2023), did not exhibit any significant alteration (Figure S4F). Together, these data indicate that *Bnip3* is a direct transcriptional target of HIF‐3α.

To further verify the regulatory hierarchy between HIF‐3α and BNIP3, we established HIF‐3α overexpression and knockdown models in HL‐1 cells. Western blot and qPCR results revealed that forced HIF‐3α overexpression markedly elevated both mRNA and protein abundance of BNIP3 (Figure 6F,G). Reciprocally, depletion of HIF‐3α suppressed BNIP3 expression (Figure 6F,H), confirming that HIF‐3α acts as a positive transcriptional regulator of BNIP3.

We next performed epistasis experiments to validate the essential role of HIF‐3α in this pathological mitophagy cascade. We overexpressed BNIP3 under HIF‐3α knockdown background and observed that the accumulation of LC3B‐II and p62 triggered by BNIP3 overexpression was partially attenuated (Figure 6I). Collectively, these data establish HIF‐3α as an indispensable upstream driver of BNIP3‐mediated mitophagy dysfunction.

### 3.7 HIF‐3α Senses Low ATP Levels in Senescent Cardiomyocytes

To explore how mitochondrial transplantation regulates the HIF‐3α‐BNIP3 axis in the aging heart, we first measured ATP production in cardiac tissues. Mitochondrial treatment markedly increased energy levels in aged cardiac tissues (Figure 6J,K), suggesting that ATP fluctuations may critically affect HIF‐3α‐BNIP3 axis activity. To verify this hypothesis, HL‐1 cells were treated with CCCP to induce mitochondrial damage and energy deficit, and HIF‐3α expression was assessed. CCCP treatment reduced ATP levels (Figure 6L) in cardiomyocytes and concomitantly upregulated HIF‐3α (Figure 6M). These results demonstrate that HIF‐3α senses low ATP levels in aged cardiomyocytes, thereby modulating BNIP3 expression and leading to mitophagy dysregulation.

Taken together, low ATP levels in the aging heart activate the HIF‐3α‐BNIP3 axis, leading to mitophagy flux dysregulation, and accelerated cardiomyocyte senescence. Furthermore, exogenous mitochondrial transplantation restores ATP production, stabilizes mitophagy flux, and thereby delays cardiac aging.

## 4 Discussion

Maintaining normal mitophagy flux is crucial for cardiomyocyte homeostasis (Fu et al. 2024; Li et al. 2016). Previous studies have established that cardiac aging is accompanied by a marked failure in mitochondrial quality control (Ravindran and Gustafsson 2025; Wu et al. 2023), consistent with the observations in the present study. However, the mechanistic linking of impaired mitochondrial quality control to accelerated cardiac aging remains unclear. Our findings demonstrate that mitophagy flux congestion in senescent cardiomyocytes leads to the accumulation of mitophagosomes, which causes mitochondrial dysfunction and accelerates cardiac aging.

Transcriptomic and proteomic sequencing in mice, together with single‐cell RNA sequencing analysis in humans, revealed significantly elevated expression of the mitophagy‐related gene *Bnip3* in aging heart. The mitophagy is primarily regulated through two pathways: the ubiquitin‐dependent PINK1/Parkin pathway and the receptor‐mediated Bnip3 pathway (Lin et al. 2021; Lu et al. 2023). We employed correlation analysis to assess their respective contributions in aged cardiac and revealed a preference for activation via the receptor‐mediated BNIP3 pathway. In vitro overexpression experiments further confirmed that excessive expression of BNIP3 directly triggers aberrant mitophagy, resulting in mitophagy flux congestion and accelerated senescence of cardiomyocytes.

HIF‐3α has long been viewed as a repressor, largely due to its short splice variants lacking the transactivation domain. However, full‐length HIF‐3α functions as an oxygen‐regulated activator, suggesting its functional outcome depends on the splice variant expressed. In the aging myocardium, the activator role of HIF‐3α is likely shaped by the local microenvironment, particularly senescence‐associated inflammation and metabolic remodeling (Cuomo et al. 2018; Diao et al. 2022). Cuomo et al. showed that HIF‐3α acts as a key regulator of energy metabolism, and that inhibiting its expression promotes white adipose tissue “browning” and energy expenditure (Cuomo et al. 2021). However, in senescent cardiomyocytes, the specific molecular mechanisms by which HIF‐3α senses or regulates ATP levels remain unclear. In this study, we found that HIF‐3α is highly sensitive to ATP levels. The fluctuations in ATP levels in senescent cardiomyocytes induce upregulation of HIF‐3α expression, which in turn affects BNIP3 expression and mitophagy. These findings may support a pathway in which energy crisis stabilizes HIF‐3α, which then upregulates BNIP3 and leads to mitophagy flux congestion. Moreover, mitochondrial transplantation could restore ATP levels in senescent cardiomyocytes and stabilize the overactivation of the HIF‐3α‐BNIP3 axis, thereby rejuvenating aging heart.

In cardiovascular research, studies have implicated HIF‐3α in the pathophysiology of various cardiac disorders, including diabetic cardiomyopathy and pathological cardiac hypertrophy (Guo et al. 2021), highlighting its significant regulatory role in cardiac homeostasis and disease progression. Its basic helix–loop–helix (bHLH) and Per/Arnt/Sim (PAS) domains, along with a trans‐activating domain, enable HIF‐3α to modulate diverse transcriptional responses under hypoxic conditions. These findings not only provide a foundation for understanding the involvement of HIF‐3α in myocardial homeostasis and aging but also suggest a potentially distinct role in the regulation of BNIP3, diverging from the canonical HIF signaling pathway. Further analysis of differentially expressed transcription factors pinpointed HIF‐3α as a putative regulator of BNIP3. Bioinformatic analyses collectively support a direct transcriptional activation: JASPAR predicted HIF‐3α binding sites in the *Bnip3* promoter, public protein atlases showed a significant expression correlation, and the HPA database provided corroborative evidence. This hypothesis was validated in cardiomyocyte, where manipulation of HIF‐3α expression (either up or down) led to corresponding changes in BNIP3 levels, establishing a clear positive regulatory relationship. Consequently, inhibiting HIF‐3α alleviated the BNIP3‐driven congestion of mitophagy flux. Strikingly, clinical data from heart failure samples reveal a concomitant upregulation of HIF‐3α and BNIP3 (Zolk, Solbach, Eschenhagen, Weidemann, and Fromm 2008), which aligns with our sequencing results. Thus, we propose that the upregulation of the HIF‐3α‐BNIP3 axis disrupts the mitophagy flux during cardiac aging. Although the GSK‐3α‐BNIP3 axis has been characterized in hypoxic mitophagy, HIF‐3α represents a distinct, transcriptionally non‐redundant regulator of BNIP3 expression (Marzook et al. 2024). We have uncovered a unique, kinase‐independent mechanism, where HIF‐3α (rather than HIF‐1α) directly activates BNIP3, resulting in abnormal mitophagy in senescent cardiomyocytes.

This study identifies that low ATP levels induce overactivation of the HIF‐3α‐BNIP3 axis, which critically drives cardiac aging. HIF‐3α upregulation induces overexpression of the mitophagy receptor BNIP3, resulting in mitophagy flux congestion characterized by increased mitophagosome formation but impaired degradation, which ultimately accelerates cardiomyocyte senescence. Notably, our mitochondria‐targeted intervention effectively normalizes HIF‐3α‐BNIP3 expression by increasing ATP production, thereby restoring mitophagy flux and mitigating cardiac aging. These findings enhance the understanding of mitochondrial quality control mechanisms in cardiac aging and provide a foundation for novel strategies targeting mitophagy flux to combat cardiac aging. While the AMPK‐PHD2 axis has been established as a key energy‐sensing pathway that regulates HIF‐3α stability through PHD2 phosphorylation, and our previous work has shown that mitochondrial transplantation exerts cardioprotection by reinstating ATP production and modulating AMPKα‐mTOR pathway (Jin et al. 2024), the precise role of AMPK in coupling ATP fluctuations to HIF‐3α stabilization in the aging heart remains to be elucidated. Future studies employing AMPK‐specific pharmacological inhibitors or genetic knockdown approaches will be necessary to dissect whether AMPK‐PHD2 signaling directly mediates the energy‐dependent regulation of HIF‐3α in senescent cardiomyocytes.

Doxorubicin induces cardiac senescence with features such as oxidative stress, mitochondrial dysfunction, and mitophagy impairment, which largely mirror the phenotypes found in naturally aged hearts and converge on the HIF‐3α‐BNIP3 axis. However, this drug‐driven model is an acute form of accelerated senescence and does not fully recapitulate the chronic, systemic nature of physiological aging, which involves multi‐organ decline and sustained inflammation. To address this, we validated our findings in 8‐week‐old versus 80‐week‐old naturally aged mice and analyzed human single‐cell datasets to assess clinical relevance. These complementary approaches support the pathophysiological significance of the HIF‐3α‐BNIP3 axis in cardiac aging.

Mitochondrial transplantation has advanced into early‐phase clinical exploration, with systematic reviews supporting its potential to restore myocardial bioenergetics (Arko et al. 2025). Phase I trials (NCT02851758) in pediatric ischemia/reperfusion injury have shown preserved cardiomyocyte viability (Emani et al. 2017). Platelet‐derived mitochondria have emerged as a promising option for ischemic heart disease (Baharvand et al. 2024). Regarding delivery, intramyocardial injection offers targeted delivery but requires invasive procedures and may compromise mitochondrial integrity, and intracoronary infusion appears most effective preclinically, while intravenous delivery is minimally invasive but faces challenges such as enzymatic degradation and low cardiac targeting efficiency (Ibanez and Villena‐Gutierrez 2021; Sun et al. 2023). Safety data are encouraging, with no immune responses reported after autologous transplantation and no tumorigenic risk (McCully et al. 2026). Nonetheless, larger controlled trials with extended follow‐up are needed to validate long‐term safety and efficacy (Baharvand et al. 2024). Given its potential therapeutic prospects (Kim et al. 2025), we evaluated the protective effect of mitochondrial transplantation on the aging heart. Our results demonstrated that transplantation significantly improved left ventricular systolic function in aged mice and downregulated the expression of senescence‐associated markers (SA‐β‐gal, p16, and APOE) in cardiac tissue. Furthermore, TEM and immunofluorescence analyses confirmed that the treatment effectively attenuated the pathological accumulation of mitophagosomes in both cardiac tissues and cardiomyocytes. Notably, BNIP3 overexpression partially abolished the protective effects of mitochondrial transplantation on mitophagy flux, mitochondrial homeostasis, and senescence delay. This confirms that mitochondrial therapy can rectify the aberrant BNIP3‐mediated mitophagy and stabilize the mitophagy flux.

This study focused on the role of the HIF‐3α‐BNIP3 axis mediated congestion of mitophagy flux in cardiac aging and the therapeutic potential of mitochondrial‐targeted intervention. However, several limitations do exist: (1) unclear mechanism of HIF‐3α sensing low ATP levels; (2) lack of direct ultrastructural evidence to confirm whether there is any physical fusion between exogenous and endogenous mitochondrial networks; (3) absence of clinical safety evaluations for mitochondrial transplantation therapy. Further research is needed to address these gaps and assess clinical feasibility.

## Author Contributions

N.J., Y.L., Z.L., and J.X. conceived the concept and designed the experiments. N.J., L.Z., and J.T. performed mitochondrial extraction and the tracing of transplantation. N.J., L.Z., H.G., J.T., G.J., H.C., X.G. H.Z., and Z.P. established the Aging cardiac mouse model, performed the animal surgeries, and conducted the in vivo therapeutic evaluations. N.J., L.Z., H.G., W.W., and H.Z. conducted cytological experiments and performed in vitro effect evaluations. N.J., Z.L., H.G., X.H., and H.C. assisted with the histological staining, flow cytometry, and Western blot analyses. N.J. and Z.L. analyzed the data and wrote the original draft of the manuscript. N.J., Y.L., Z.L. and J.X. supervised the study and reviewed and edited the manuscript. All authors discussed the results and approved the final version of the manuscript.

## Funding

The work was supported by the National Nature Science Foundation of China[U23A20420] (J.X.) and Shanxi Province Higher Education “Billion Project” Science [BYBLD006] (J.X.).

## Conflicts of Interest

The authors declare no conflicts of interest.

## Supporting information


**Figure S1:** Transplantation of BMSC‐derived mitochondria into cardiac tissue and HL‐1 cells. (A) In vivo imaging of mice at 1, 2, 3, 5, 8, 10, 12, and 24 h after mitochondrial transplantation. Signal intensity is indicated by the color scale. (B) Live cell imaging after mitochondrial transplantation in HL‐1 cells. The arrow represents the process of mitochondria entering the cell. (C) Particle size distribution of isolated mitochondria measured by dynamic light scattering. The measured diameters fell predominantly within the range of 500–1000 nm, which is consistent with the reported size range of intact mitochondria. (D) Mitochondrial membrane potential (ΔΨm) assessed by JC‐1 staining, presented as the ratio of 590 nm (aggregates) to 530 nm (monomers) fluorescence. Data are shown as mean ± SD from three independent experiments (*n* = 3). (E) Intracellular ATP luminescence value in aged cardiomyocytes with or without mitochondrial treatment over a 48 h time course. Two‐way repeated‐measures ANOVA: time effect, *p* < 0.0001; treatment effect, *p* < 0.0001; time × treatment interaction, *p* = 0.0126. (F) Intracellular ATP luminescence value in young cardiomyocytes with or without mitochondrial treatment over a 48‐h time course. Two‐way repeated‐measures ANOVA: time effect, *p* < 0.0001; treatment effect, *p* = 0.0223; time × treatment interaction, *p* = 0.9231. (G) Quantification of intracellular ATP luminescence value at early time points (3, 6, and 9 h) in aged and young cardiomyocytes with or without mitochondrial treatment. Paired Student's *t*‐test: Data are shown as mean ± SD. *p < 0.05, **p < 0.01, ***p < 0.001 versus corresponding untreated group.
**Figure S2:** Expression analysis of mitophagy‐related proteins. (A, B) Expression and quantitative analysis of BNIP3 in aged cardiac tissue and senescent HL‐1 cells by Western blot analysis (*n* = 3 per group). (C, D) Expression and quantitative analysis of Parkin proteins (*n* = 3 per group). (E) Analysis of mitochondrial‐related DEmRNAs in aged and aged+mito groups. The expression values were represented by a color scale. A1, A2, A3: aged tissue, B1, B2, B3: aged+mito tissue. (F) Expression of *Bnip3* mRNA in cardiac tissue (*n* = 3 per group). (G) GSEA on the “TABULA_MURIS_SENIS_HEART_AND_AORTA_ CARDIOMYOCYTE_AGEING” gene set. Bars represent mean ± SD. *p < 0.05, **p < 0.01, ***p < 0.001 Statistical significance was assessed using an unpaired two‐tailed Student's *t*‐test.
**Figure S3:** The expression of BNIP3. (A) Representative immunohistochemistry images of BNIP3 in normal human cardiac tissues from the HPA database. (B, C) Representative fluorescence images of BNIP3 in HL‐1 cells and quantitative analysis of fluorescence intensity (*n* = 3 per group). Scale bars, 100 μm. (D) Expression and quantification of BNIP3 after transfecting with PEX‐1 (control) or OE‐*Bnip3* plasmids for 24 h by Western blot analysis (*n* = 3 per group). (E) Analysis of *Bnip3* mRNA expression by RT‐qPCR after transfecting with PEX‐1 (control) or OE‐Bnip3 plasmids for 24 h (*n* = 3 per group). (F) Analysis of Map1lc3b and *p62* mRNA expression by RT‐qPCR after transfecting with PEX‐1 or OE‐Bnip3 plasmids for 24 h (*n* = 3 per group). (G, H) Expression and quantification of p16 and APOE after transfecting with PEX‐1 or OE‐*Bnip3* plasmids for 24 h by Western blot analysis (*n* = 3 per group). (I, J) SA‐β‐Gal staining and quantitative analysis of HL‐1 cells transfected with PEX‐1 or OE‐*Bnip3* plasmids for 24 h. (*n* = 3 per group). Scale bars, 100 μm. Comparisons between two groups were performed using the unpaired *t*‐test. For comparisons among multiple groups, one‐way analysis of variance (ANOVA) was used. Bars represent mean ± SD. *p < 0.05, **p < 0.01, ***p < 0.001.
**Figure S4:** Expression of HIF‐3α and the Correlation with BNIP3. (A) Correlation analysis of HIF‐3α and BNIP3 via the GEPIA2 platform. (B) Representative immunohistochemistry images of HIF‐3α in normal human cardiac tissues from the HPA database. (C) UMAP gene expression visualization showed the expression of HIF‐3α. Cell type annotation is shown in Figure 4K. (D) Violin plot quantitative analysis of the expression levels of HIF‐3α in cardiomyocytes. (E, F) Expression and quantification of HIF‐3α protein in the cardiac tissues of naturally aged mice by Western blot (*n* = 3 per group). (G) Statistical analysis of *BNIP3* mRNA expression in the cardiac tissues of naturally aged mice (*n* = 3 per group). (H) Expression of HIF‐1α in HL‐1 cells by Western blot analysis. (I) KEK293T cells were cotransfected with the *BNIP3* reporter and the HIF‐3α plasmids for 24 h. Luciferase activity was then detected by a dual‐luciferase assay. (J, K) Representative fluorescence images of HIF‐3α and the quantitative analysis of fluorescence intensity in HL‐1 cells (*n* = 3 per group), Scale bars, 100 μm. Comparisons between two groups were performed using the unpaired *t*‐test. For comparisons among multiple groups, one‐way analysis of variance (ANOVA) was used. Bars represent mean ± SD. *p < 0.05, **p < 0.01, ***p < 0.001.
**Table S1:** Antibodies used for western blotting and/or immunofluorescence staining.
**Table S2:** Primer sequences for genes analyzed by RT‐qPCR.
**Table S3:** The primers sequences for ChIP‐PCR.

**Movie S1:** Video of mitochondrial transplantation in ex vivo.

## Data Availability

All data needed to evaluate the conclusions in the paper are present in the paper and/or the Supporting Information. The raw data files of RNA‐seq data generated in this study have been deposited and are available in the Genome Sequence Archive (GSA) of the National Genomics Data Center (NGDC, China National Center for Bioinformation/Beijing Institute of Genomics, Chinese Academy of Sciences) (CRA038101) that are publicly accessible at https://ngdc.cncb.ac.cn/gsa. The raw proteomic data are available in OMIX database (https://ngdc.cncb.ac.cn/omix) with accession ID OMIX019861.
