# Supplementary material for: Mitochondrial Transplantation Rejuvenates Aging Heart by Restoring Mitophagy Flux via the HIF‐3α‐BNIP3 Axis
Source: Aging Cell. 2026 Sep 23;25(10):e70720. doi: 10.1111/acel.70720 (PMC13599351; doi:10.1111/acel.70720)
Supplement: Supplementary file 1 [file ACEL-25-e70720-s001.docx]

**Mitochondrial Transplantation Rejuvenates Aging Heart by Restoring Mitophagy Flux via the Hif3α-Bnip3 Axis**

Ning Jin *et al.*

**This PDF file includes:**

Supplementary figures and figure legends (S1 to S4)

Tables S1 to S3

Movie S1

Supplementary figures and figure legends

Figure S1. Transplantation of BMSC-derived mitochondria into cardiac tissue and HL-1 cells. (A) In vivo imaging of mice at 1, 2, 3, 5, 8, 10, 12, and 24 hours after mitochondrial transplantation. Signal intensity is indicated by the color scale. (B) Live cell imaging after mitochondrial transplantation in HL-1 cells. The arrow represents the process of mitochondria entering the cell. (C) Particle size distribution of isolated mitochondria measured by dynamic light scattering. The measured diameters fell predominantly within the range of 500-1000 nm, which is consistent with the reported size range of intact mitochondria. (D) Mitochondrial membrane potential (ΔΨm) assessed by JC‑1 staining, presented as the ratio of 590 nm (aggregates) to 530 nm (monomers) fluorescence. Data are shown as mean ± SD from three independent experiments (n = 3). (E) Intracellular ATP luminescence value in aged cardiomyocytes with or without mitochondrial treatment over a 48h time course. Two‑way repeated‑measures ANOVA: time effect, P < 0.0001; treatment effect, P < 0.0001; time × treatment interaction, P = 0.0126. (F) Intracellular ATP luminescence value in young cardiomyocytes with or without mitochondrial treatment over a 48‑h time course. Two‑way repeated‑measures ANOVA: time effect, P < 0.0001; treatment effect, P = 0.0223; time × treatment interaction, P = 0.9231. (G) Quantification of intracellular ATP luminescence value at early time points (3, 6, and 9 h) in aged and young cardiomyocytes with or without mitochondrial treatment. Paired Student's t‑test : Data are shown as mean ± SD. *P < 0.05, **P < 0.01, ***P < 0.001 vs. corresponding untreated group.

**Figure S2. Expression analysis of mitophagy-related proteins.** (A-B) Expression and quantitative analysis of BNIP3 in aged cardiac tissue and senescent HL-1 cells by Western blot analysis (n=3 per group). (C-D) Expression and quantitative analysis of Parkin proteins (n=3 per group). (E) Analysis of mitochondrial-related DEmRNAs in aged and aged+mito groups. The expression values were represented by a color scale. A1, A2, A3: aged tissue, B1, B2, B3: aged+mito tissue. (F) Expression of *Bnip3* mRNA in cardiac tissue (n=3 per group). (G) GSEA on the “TABULA_MURIS_SENIS_HEART_AND_AORTA_ CARDIOMYOCYTE_AGEING” gene set. Bars represent mean ± SD. *P < 0.05, **P < 0.01, ***P < 0.001 Statistical significance was assessed using an unpaired two-tailed Student’s t-test.

Figure S3. The expression of Bnip3. (A) Representative immunohistochemistry images of BNIP3 in normal human cardiac tissues from the HPA database. (B-C) Representative fluorescence images of BNIP3 in HL-1 cells and quantitative analysis of fluorescence intensity (n=3 per group). Scale bars, 100μm. (D) Expression and quantification of BNIP3 after transfecting with PEX-1 (control) or OE-*Bnip3* plasmids for 24h by Western blot analysis (n=3 per group). (E) Analysis of *Bnip3* mRNA expression by RT-qPCR after transfecting with PEX-1 (control) or OE-Bnip3 plasmids for 24 h (n=3 per group). (F) Analysis of Map1lc3b and *p62* mRNA expression by RT-qPCR after transfecting with PEX-1 or OE-Bnip3 plasmids for 24 h (n=3 per group). (G-H) Expression and quantification of p16 and APOE after transfecting with PEX-1 or OE-*Bnip3* plasmids for 24h by Western blot analysis (n=3 per group). (I-J) SA-β-Gal staining and quantitative analysis of HL-1 cells transfected with PEX-1 or OE-*Bnip3* plasmids for 24h. (n=3 per group). Scale bars, 100μm. Comparisons between two groups were performed using the unpaired t-test. For comparisons among multiple groups, one-way analysis of variance (ANOVA) was used. Bars represent mean ± SD. *P < 0.05, **P < 0.01, ***P < 0.001

**Figure S4. Expression of Hif‑3α and the Correlation with Bnip3.** (A) Correlation analysis of HIF-3α and BNIP3 via the GEPIA2 platform. (B) Representative immunohistochemistry images of HIF-3α in normal human cardiac tissues from the HPA database. (C) UMAP gene expression visualization showed the expression of HIF-3α. Cell type annotation is shown in Figure 4K. (D) Violin plot quantitative analysis of the expression levels of HIF-3α in cardiomyocytes. (E-F) Expression and quantification of HIF-3α protein in the cardiac tissues of naturally aged mice by Western blot (n=3 per group). (G) Statistical analysis of *BNIP3* mRNA expression in the cardiac tissues of naturally aged mice (n=3 per group). (H) Expression of HIF-1α in HL-1 cells by Western blot analysis. (I) KEK293T cells were cotransfected with the *BNIP3* reporter and the HIF-3α plasmids for 24 h. Luciferase activity was then detected by a dual-luciferase assay. (J-K) Representative fluorescence images of HIF-3α and the quantitative analysis of fluorescence intensity in HL-1 cells (n=3 per group), Scale bars, 100μm. Comparisons between two groups were performed using the unpaired t-test. For comparisons among multiple groups, one-way analysis of variance (ANOVA) was used. Bars represent mean ± SD. *P < 0.05, **P < 0.01, ***P < 0.001

Supplementary Tables

Table S1. Antibodies used for western blotting and/or immunofluorescence staining.

| Antibody | Vendor | Catalog Number | Application Working | Dilution for WB or IF |
| --- | --- | --- | --- | --- |
| Rabbit anti-GAPDH | Proteintech | 10494-1-AP | WB | 1: 10000 |
| Beta Actin Monoclonal antibody | Proteintech | 66009-1-Ig | WB | 1: 10000 |
| APOE Rabbit pAb | ABclonal | A16344 | WB | 1: 1000 |
| CDKN2A/p16^Ink4a^ | Wanlei | WL01418 | WB | 1: 1000 |
| LC3B Antibody | Abmart | T55992 | WB,IF | 1: 1000, 1: 200 |
| Anti-p62 Rabbit pAb | Wanlei | WL02385 | WB | 1: 1000 |
| VDAC1/Porin Polyclonal antibody | Proteintech | 55259-1-AP | WB | 1: 1000 |
| Parkin Antibody | Abmart | T56641 | WB | 1: 1000 |
| Anti-BNIP3 antibody | Abcam | AB109362 | WB,IF | 1: 1000, 1: 200 |
| HIF-3α Polyclonal antibody | Proteintech | 27650-1-AP | WB,IF | 1: 1000, 1: 200 |
| Goat Anti-Rabbit IgG H&L, HRP conjugated | BIOSS | bs-0295G-HRP | WB | 1: 10000 |
| Multi-rAb™ HRP-Goat Anti-Mouse Recombinant Secondary Antibody (H+L) | Proteintech | RGAM001 | WB | 1: 8000 |
| Goat Anti-Rabbit IgG H&L, BF488 conjugated | BIOSS | bs-0295G-BF488 | IF | 1: 200 |

Table S2. Primer sequences for genes analyzed by RT-qPCR.

| **Gene** | **Primer** |
| --- | --- |
| *β-actin* | F: 5'-CGTTGACATCCGTAAAGACC-3' |
|  | R: 5'-AACAGTCCGCCTAGAAGCA-3' |
| *Bnip3* | F: 5'-GCGTTCCAGCCTCCGTCTC-3' |
|  | R: 5'-CTCTGTTGGTATCTTGTGGTGTCTG-3' |
| *Hif3α* | F: 5'-AATGCCTGGTGCTTATCTGTGAAG-3' |
|  | R: 5'-CAATCCTCTCGTCGCAGTATGTG-3' |
| *Parkin* | F: 5'-CTATAAGATGGTGGGCACCGTGATC-3' |
|  | R: 5'-CTCCTTGAACTCCTCCAGCGTAATC-3' |
| *Sqstm1* | F: 5'-AGCACAGGCACAGAAGACAAGAG-3' |
|  | R: 5'-TCCCACCGACTCCAAGGCTATC-3' |

**Table S3. The primers sequences for ChIP-PCR**

| **Gene** | **Primer** |
| --- | --- |
| Site1*-Bnip3-*1 | F: GTGCCACACGCTCCCCCGCG |
|  | R:GAGGGACAAGGGGCGGGGCG |
| Site1*-Bnip3-*2 | F: CCCCGCCCCCCAGCCAATGG |
|  | R: R:GACAAGGGGCGGGGCGGGG |
| Site2*-Bnip3-*1 | F: TGCCTCAGGCCCCGCCCATG |
|  | R:AACGCGGGGGAGCGTGTGGC |
| Site2*-Bnip3-*2 | F: CAGCCGCCCTCCCGCGCACT |
|  | R:GCCTGCAGGTGGGGGAGGAA |

Movie S1. Video of mitochondrial transplantation in ex vivo.
